# Radiation‐induced mesothelioma among long‐term solid cancer survivors: a longitudinal analysis of SEER database

**DOI:** 10.1002/cam4.656

**Published:** 2016-02-10

**Authors:** Andrea Farioli, Marta Ottone, Alessio G. Morganti, Gaetano Compagnone, Fabrizio Romani, Silvia Cammelli, Stefano Mattioli, Francesco S. Violante

**Affiliations:** ^1^ Department of Medical and Surgical Sciences (DIMEC)University of BolognaBolognaItaly; ^2^ Deptartments of ExperimentalDiagnostic and Specialty Medicine ‐ DIMES Radiation Oncology CenterUniversity of BolognaS.Orsola‐Malpighi HospitalBolognaItaly; ^3^ Department of Medical PhysicsS.Orsola‐Malpighi University HospitalBolognaItaly

**Keywords:** Cohort study, dose–response relationship, radiation, mesothelioma, radiation‐induced malignancies, radiotherapy, SEER program

## Abstract

We investigated the association between external beam radiotherapy (EBRT) and pleural and peritoneal mesothelioma among long‐term (>5 years) solid cancer survivors. We analyzed data from the US Surveillance, Epidemiology, and End Results (SEER) program (1973–2012). We fitted survival models adjusted by age, gender, race, year, surgery, and relative risk of primary mesothelioma in the county of residence (proxy for individual asbestos exposure). We estimated hazard ratios [HR] with reference to nonirradiated patients. We distinguished between scattered and direct irradiation to study the dose–response. We observed 301 mesotheliomas (265 pleural; 32 peritoneal; 4 others) among 935,637 patients. EBRT increased the risk of mesothelioma (any site; HR 1.34, 95% CI 1.04–1.77). We observed an increased risk of pleural mesothelioma (HR for EBRT 1.34, 95% CI 1.01–1.77), but we did not find signs of a dose–response relationship (HR for scattered irradiation 1.38; HR for direct irradiation 1.23). On the opposite, only direct peritoneal irradiation was associated with peritoneal mesothelioma (HR 2.20, 95% CI 0.99–4.88), particularly for latencies ≥10 years (HR 3.28, 95% CI 1.14–9.43). A competing risks analysis revealed that the clinical impact of radiation‐induced mesothelioma was limited by the high frequency of competing events. The cumulative incidence function of mesothelioma after 40 years of observation was very low (nonirradiated patients 0.00032, irradiated patients 0.00055).EBRT might be a determinant of mesothelioma. Longer latency periods are associated with higher risks, while the dose–response seems nonlinear. The clinical impact of mesothelioma after EBRT for primary solid cancers is limited.

## Introduction

Malignant mesothelioma is a rare cancer usually originating from the lining cells of the pleural and peritoneal cavities 1. Asbestos is by far the most important risk factor for mesothelioma; however, a background lifetime probability (i.e., the risk of getting the disease in the absence of exposure to asbestos) of about 3 per 10,000 has been estimated 2, 3. Hence, recent experimental and epidemiological studies have focused on other potential causal factors of mesothelioma, including: nonasbestiform mineral fibers (erionite; fluoro‐edenite); carbon nanotubes; viruses (MC29 avian leukosis virus; SV40); metals; chronic serosal inflammation; and ionizing radiation 4.

The association between ionizing radiation and mesothelioma has been studied among nuclear power plant workers and among patients exposed to the diagnostic X‐ray contrast medium ‘‘Thorotrast’’ or to external beam radiotherapy (EBRT) 5. However, available evidence on the association between EBRT and mesothelioma is still controversial 5. First, most studies were based on a limited number of mesothelioma cases. Second, there is lack of knowledge on the possible dose–response relationship. In a previous study, we reported an increased incidence of mesothelioma after EBRT for prostate cancer 6. Remarkably, we found suggestive evidence that the magnitude of the risk could depend on the distance of the mesothelium from the irradiated field. Indeed, we observed the higher relative risk for peritoneal mesothelioma, which occurs within the irradiated field 6.

To provide further evidence of the association between EBRT and mesothelioma and to fill the knowledge gap on the dose–response, we conducted an analysis of mesothelioma incidence after EBRT for primary solid cancer using data from the from the US National Cancer Institute's Surveillance, Epidemiology, and End Results (SEER) Program. We also aimed at evaluating the clinical impact of radiation‐induced mesotheliomas among EBRT patients.

## Materials and methods

### Study population and follow‐up

We defined the cohort as adult (age>20 years) patients with a first primary solid cancer reported to one of the SEER registries. The SEER 9 Registries database was consulted for the period between Jan 1, 1973 and Dec 31, 1991, while the SEER 13 Registries database was used for the period between Jan 1, 1992 and Dec 31, 2012. We studied only cancer sites that were frequently (>10%) treated with EBRT. Because of the minimum latency period for radiation‐induced solid neoplasms, we excluded subjects who survived <5 years after the primary diagnosis and we started the follow‐up window after the fifth year of survival 7. We removed from the cohort patients with a diagnosis of bone, soft tissue, nerve or “other endocrine” cancers because of uncertainty on the irradiated areas. Moreover, we excluded subjects with primary cancers of pleura, peritoneum, retroperitoneum, omentum, or other mesentery due to the possibility that these neoplasms were misdiagnosed mesotheliomas. We applied listwise deletion and we analyzed only subjects with complete information on radiotherapy, surgery, and county of residence. In this study, we did not consider subjects who had received radiotherapy treatments other than EBRT. On the one hand, this group could not be merged to the reference category (nonirradiated subjects) because there is not definitive evidence that these treatments are not associated with an increased risk of second cancer, for example,^8^. On the other hand, the number of cases (*n *= 20) observed was too limited to conduct separate analyses. Finally, we excluded those cancer sites with less than one expected case of mesothelioma (estimated based on the calendar year‐, age‐, sex‐ and race‐specific mesothelioma rates observed in the SEER Registries database; see Table S1). A list of the studied primary cancer sites is presented in Table 1 alongside with the number of observed mesothelioma cases.

**Table 1:** Number of mesothelioma cases and classification of radiation dose to pleura and peritoneum based on primary cancer site

	Mesothelioma cases			Dose	
Primary cancer site	Pleural (*n *= 265)	Peritoneal (*n* = 32)	Other/unknown (*n* = 4)	Pleura	Peritoneum
Eye and orbit	2	0	0	Scattered	Scattered
Oral cavity and pharynx	5	1	0	Direct	Scattered
Larynx	7	0	0	Direct	Scattered
Lung and bronchus	4	1	0	Direct	Scattered
Breast	27	8	0	Direct	Scattered
Stomach	2	0	0	Direct	Direct
Rectum and rectosigmoid junction	19	2	0	Scattered	Direct
Cervix uteri	1	1	0	Scattered	Direct
Corpus uteri and uterus NOS	7	4	0	Scattered	Direct
Prostate	186	14	4	Scattered	Direct
Testis	5	1	0	Scattered	Direct
Penis and other male genital organs	0	0	0	Scattered	Direct

NOS, not otherwise specified.

The follow‐up time for each individual started 5 years after the first primary diagnosis and ended at the diagnosis of mesothelioma, incidence of a second malignancy other than mesothelioma, death, or at the end of the study (12/31/2012). Death or incidence of a second malignancy other than mesothelioma were treated as competing events and subjects lost to follow‐up were right‐censored. Also, we censored the follow‐up at the age of 85 years due to the known under‐ascertainment of second primary cancers after that age in the SEER registries 9.

This study was conducted in compliance with the 1964 Helsinki declaration and its later amendments. Given the retrospective nature of this study conducted using register data, formal consent was not required.

### Outcome measures and case definitions

We investigated the risk of mesothelioma occurring in any site, of pleural and peritoneal mesothelioma. As many patients of our cohort died during the study period or developed a second malignancy other than mesothelioma, we accounted for competing risks in our analysis 10. To answer our main etiological research question on the causal association between EBRT and mesothelioma, we estimated the cause‐specific hazard ratio (HR) of mesothelioma 10, 11, 12. Then, to evaluate the clinical impact of the observed associations, we modeled the subhazard ratio [SHR] and the cumulative incidence function (CIF) of mesothelioma 10, 11, 12.

### Exposure and covariates

Patients were classified according to whether or not they had received radiotherapy as a part of their initial treatment for primary cancer. We compared patients who received EBRT (alone or in combination with other forms of radiotherapy) with those who were not treated with any form of radiotherapy. When studying specific mesothelioma sites, we further classified the exposure based on the presumed dose received by the mesothelium (see Table 1) 13, 14. We created the following three categories of exposure to radiation:

1. unexposed, did not receive any radiotherapy;
2. scattered exposure, primary cancer located far (≥3 cm) from the pleura and thoracic/cervical lymph nodes unlikely to be irradiated during the initial treatment (study of pleural mesothelioma), or primary cancer located far from the peritoneum (study of peritoneal mesothelioma);
3. direct, primary cancer located next (<3 cm) or within the pleura and/or thoracic/cervical lymph nodes likely to be irradiated (study of pleural mesothelioma), or primary cancer located next or within the peritoneum (study of peritoneal mesothelioma).

Covariates to be included in the multivariable models were selected a priori and included: age, sex, calendar year, race (white, black, other), and surgery of primary cancer.

No individual information is available in the SEER database on the exposure to asbestos, the main determinant of mesothelioma 1. As the uses of asbestos tended to be clustered and pleural mesothelioma mortality at the population level has been proposed as a suitable indicator of asbestos exposure 15, 16 we conducted a spatial analysis to derive a proxy exposure variable. At first, we estimated the relative risk (RR) of primary mesothelioma among males – the fraction of cases attributable to asbestos among females is lower 1, 17) – by county using the SEER 13 Registries data (1992–2012). Then, we classified each subject according to the county's RR of mesothelioma, based on the county of residence at the time of the primary diagnosis.

Latency (time since first exposure) was considered as a possible effect modifier of the relationship between EBRT and mesothelioma because the risk of cancer increases for decades after radiation exposure 7, 13. Latency was calculated with reference to the date of the primary diagnosis and grouped into two categories: 5–10 years; more than 10 years.

### Statistical analysis

In the descriptive tables, continuous variables were expressed as mean and standard deviation (SD), whereas categorical variables were summarized as number and percentages.

Using survival time as the main temporal axis, we fitted Cox proportional hazards regression models to estimate cause‐specific HR and Fine and Gray competing risks regression models to estimate SHR 18. We additionally adapted regression models including product terms between the exposure of interest and the latency period, but due to the limited number of peritoneal mesothelioma cases among subjects who received scattered radiation (*n* = 3), this category was not retained in this analysis. We estimated the CIF of mesothelioma in any site through flexible parametric survival models for competing risk 19. In these models, three separate baseline hazards (three degrees of freedom each) were allowed to model mesothelioma, other malignancies, and death incidence. The exposure variables of interest were included in the models as time varying covariates (three degree of freedom) and we allowed for a different effect of the covariates across the competing events. All the analyses were adjusted by sex and age (parameterized as age and squared age). When investigating the risk of any mesothelioma and pleural mesothelioma, we further conducted multivariable regression models additionally adjusted by race (white, black, or other), year of primary cancer diagnosis, primary cancer surgery, and county's mesothelioma RR. Fully adjusted HR were not estimated for peritoneal mesothelioma due to the limited number of analyzed cases. As a sensitivity analysis, we fitted a series of bivariate regression models including the variable for the peritoneal dose and the potential confounders one at time.

We fitted an additional set of Cox regression models assuming shared‐frailty (gamma‐distributed latent random effects) by primary cancer site to model within‐group correlation. However, the theta parameter for random effects never approached the statistical significance (*P *< 0.05) and the estimates for the fixed part of the models (i.e., the HR) were not materially changed by the inclusion of the random parameters. Hence, our final models did not assume shared‐frailty by primary cancer.

The RR of mesothelioma by county were estimated using the Besag–York–Mollie (BYM) model, which allows for both heterogeneous and spatially structured random effects 20. Additional details on the BYM model are presented in Supplementary Resource 1 alongside with maps showing the distribution of standardized incidence ratios and RR (Fig. S1 and 2).

Figures on cancer and individual records were obtained using SEER*Stat software 8.2.1. We used Stata 12.1 SE (Stata Corporation, Texas, TX) software package for the main analysis.

## Results

**Figure 1:**
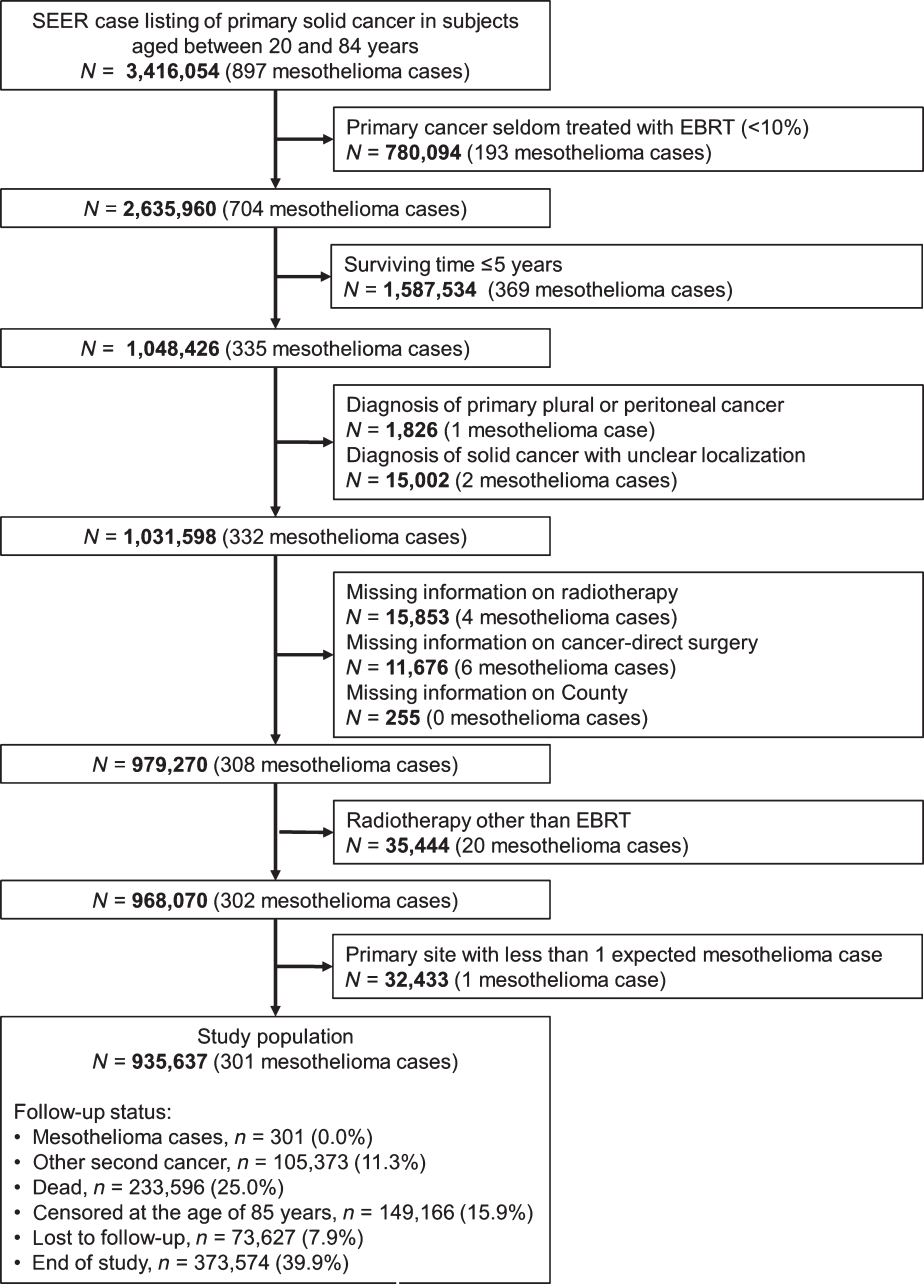
Flowchart of the study population. Patients affected by primary cancer followed up for malignant mesothelioma. EBRT, external beam radiotherapy.

As shown in Figure 1, the SEER registries included 3,416,054 cases of primary cancer among subjects aged between 20 and 84 years. In the present analysis, we considered only frequently irradiated sites with at least one expected mesothelioma case during the study period, including: eye or orbit; oral cavity and pharynx; larynx; lung and bronchus; breast; stomach; rectum and rectosigmoid junction; cervix uteri; corpus uteri and uterus not otherwise specified; prostate; testis; penis and other male genital organs. After exclusion of subjects with missing information on radiotherapy, surgery or county, or who had received radiotherapy other than EBRT, we identified a cohort of 935,637 patients that entered the main analysis. We observed 301 incident mesothelioma cases in the study population: 265 pleural, 32 peritoneal, and 4 cases in other or unknown sites.

Diagnostic techniques used to confirm the diagnosis of mesothelioma were: histology (*n* = 263, 87.4%); exfoliative cytology (*n* = 28, 9.3%); direct visualization (*n* = 1, 0.3%); radiography (*n* = 6, 2.0%); and clinical diagnosis (*n* = 2, 0.7%). Quality of diagnosis was unknown for one case (0.3%).

A summary of the cohort (Table 2) reveals differences in use of EBRT by genders, calendar periods, and cancer‐direct surgery. The use of EBRT was more frequent among females and increased over time. Also, irradiation was more common among nonoperated patients. Table S2 presents the characteristics of the study population by primary cancer site. In our cohort, the percentage of irradiated patients ranged between 15.1% (stomach) and 73.8% (larynx).

**Table 2:** Characteristics of the study population at the diagnosis of primary cancer

Characteristic	External beam radiotherapy			
	No		Yes	
	(*n *= 593,949)		(*n *= 341,688)	
Age (years), mean (SD)	60.1	(12.2)	59.7	(12.2)
Gender				
Female, *n* (%)	308,045	(61.2)	195,139	(38.8)
Male, *n* (%)	285,904	(66.1)	146,549	(33.9)
Race				
White, *n* (%)	494,587	(63.8)	280,959	(36.2)
Black, *n* (%)	51,761	(62.1)	31,564	(37.9)
Other, *n* (%)	47,601	(62.0)	29,165	(38.0)
Year of diagnosis				
1973–1977, *n* (%)	45,117	(75.4)	14,722	(24.6)
1978–1982, *n* (%)	50,548	(74.0)	17,803	(26.0)
1983–1987, *n* (%)	59,044	(70.3)	24,981	(29.7)
1988–1992, *n* (%)	85,077	(66.8)	42,298	(33.2)
1993–1997, *n* (%)	116,369	(62.7)	69,110	(37.3)
1998–2002, *n* (%)	118,052	(57.4)	87,593	(42.6)
2003–2007, *n* (%)	119,742	(58.4)	85,181	(41.6)
Cancer‐direct surgery				
no, *n* (%)	72,073	(41.0)	103,594	(59.0)
yes, *n* (%)	521,876	(68.7)	238,094	(31.3)
County's mesothelioma relative risk				
<0.67	64,072	(66.1)	32,792	(33.9)
0.67–0.90	232,655	(65.2)	124,213	(34.8)
0.91–1.09	126,269	(63.6)	72,204	(36.4)
1.10–1.49	147,659	(60.0)	98,416	(40.0)
≥1.50	23,294	(62.4)	14,063	(37.6)
Mesothelioma				
no, *n* (%)	593,762	(63.5)	341,574	(36.5)
yes, *n* (%)	187	(62.1)	114	(37.9)

Table 3 presents the cause‐specific HR of mesothelioma. We observed an increased risk of mesothelioma in any site among EBRT patients compared to nonirradiated subjects (fully adjusted HR = 1.34, 95% CI 1.04–1.74) and the estimate was higher after a minimum latency period of 10 years (fully adjusted HR = 1.58, 95% CI 1.10–2.26). The risk of pleural mesothelioma was higher among irradiated subjects (fully adjusted HR for EBRT = 1.34, 95% CI 1.01–1.77), but there were no important differences between radiation doses (fully adjusted HR: scattered irradiation = 1.38, 95% CI 1.01–1.89; direct irradiation = 1.23, 95% CI 0.77–1.96). Again, the associations were stronger after 10 years from the irradiation (fully adjusted HR for EBRT = 1.49, 95% CI 1.00–2.21). When investigating peritoneal mesothelioma, we found clear signs of an association with EBRT only for direct irradiation (age‐ and sex‐adjusted HR = 2.20, 95% CI 0.99–4.88; HR for latency periods of more than 10 years = 3.28, 95% CI 1.14–9.43). As shown in Table S3, bivariate regression models revealed that age and sex were the most relevant confounders in the association between EBRT and peritoneal mesothelioma; furthermore, the other potential confounders selected a priori did not materially change the estimates for EBRT (Table S3).

**Table 3:** Cause‐specific hazard ratios of mesothelioma after external beam radiation therapy

	All latency periods							Latency periods between 5 and 10 years^a^						Latency periods of more than 10 years^a^							
	Case		Age‐ and sex‐adjusted estimates			Fully adjusted estimates^b^		Case		Age‐ and sex‐adjusted estimates		Fully adjusted estimates^b^		Case			Age‐ and sex‐adjusted estimates			Fully adjusted estimates^b^	
Site/exposure	Yes	No	HR		(95% CI)	HR	(95% CI)	Yes	No	HR	(95% CI)	HR	(95% CI)	Yes		No	HR		(95% CI)	HR	(95% CI)
Any site																					
EBRT																					
No	187	593,762	1.00		(Ref.)	1.00	(Ref.)	102	593,847	1.00	(Ref.)	1.00	(Ref.)		85	329,786		1.00	(Ref.)	1.00	(Ref.)
Yes	114	341,574	1.29		(1.02–1.63)	1.34	(1.04–1.74)	62	341,626	1.13	(0.82–1.55)	1.17	(0.84–1.65)		52	173,686		1.52	(1.08–2.15)	1.58	(1.10–2.26)
Pleura																					
EBRT																					
No	166	593,783		1.00	(Ref.)	1.00	(Ref.)	91	593,858	1.00	(Ref.)	1.00	(Ref.)	75		329,796		1.00	(Ref.)	1.00	(Ref.)
Yes	99	341,589		1.27	(0.99–1.63)	1.34	(1.01–1.77)	57	341,631	1.17	(0.84–1.63)	1.23	(0.86–1.76)	42		173,696		1.42	(0.97–2.07)	1.49	(1.00–2.21)
Pleural irradiation																					
None	166	593,783		1.00	(Ref.)	1.00	(Ref.)	91	593,858	1.00	(Ref.)	1.00	(Ref.)	75		329,796		1.00	(Ref.)	1.00	(Ref.)
Scattered	77	150,507		1.28	(0.97–1.69)	1.38	(1.01–1.89)	46	150,538	1.19	(0.83–1.70)	1.28	(0.87–1.89)	31		74,446		1.43	(0.94–2.18)	1.53	(0.99–2.38)
Direct	22	191,082		1.22	(0.77–1.94)	1.23	(0.77–1.96)	11	191,093	1.10	(0.58–2.09)	1.09	(0.57–2.08)	11		99,250		1.37	(0.72–2.61)	1.40	(0.73–2.68)
*P trend*					0.096		0.085				0.426		0.388						0.103		0.087
Peritoneum																					
EBRT																					
No	19	593,930		1.00	(Ref.)	/c	/c	10	593,939	1.00	(Ref.)	/c	/c	9		329,862		1.00	(Ref.)	/c	/c
Yes	13	341,675		1.41	(0.70–2.87)	/c	/c	4	341,684	0.72	(0.23–2.30)	/c	/c	9		173,729		2.31	(0.91–5.83)	/c	/c
Peritoneal irradiation																					
None	19	593,930		1.00	(Ref.)	/c	/c	10	593,939	1.00	(Ref.)	/c	/c	9		329,862		1.00	(Ref.)	/c	/c
Scattered	3	190,044		0.64	(0.18–2.21)	/c	/c	0	190,047	/d	/d	/c	/c	3		98,979		/d	/d	/c	/c
Direct	10	151,631		2.20	(0.99–4.88)	/c	/c	4	151,637	1.39	(0.42–4.53)	/c	/c	6		74,750		3.28	(1.14–9.43)	/c	/c
*P trend*					0.114																

95% CI, 95% confidence intervals; EBRT, external beam radiotherapy; HR, hazard ratio; Ref., reference category.

a Estimates from regression models including an interaction term between latency and EBRT.

b Estimates adjusted by age, sex, race, year of primary cancer diagnosis, primary cancer surgery, and county's mesothelioma relative risk.

c Analysis not performed due to the limited number of events.

d This category was excluded due to the limited number of cases.

**Figure 2:**
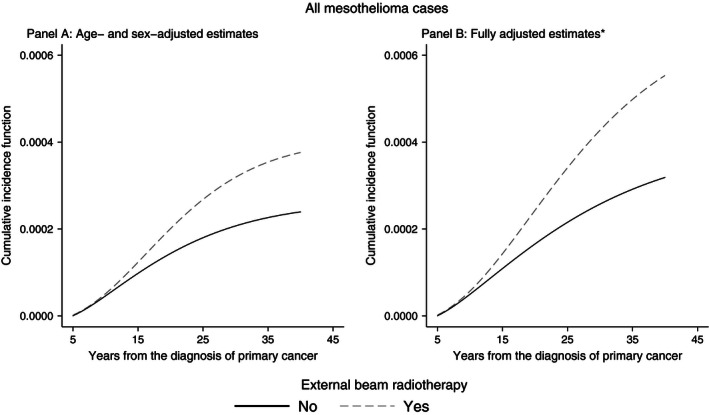
Cumulative incidence function of mesothelioma in any site; estimates from flexible parametric survival models for competing risks. *Cumulative incidence functions adjusted by age (60 years, mean value), sex (female, modal value), race (white, modal value), year of primary cancer diagnosis (1996, median value), primary cancer surgery (performed, modal value), and county's mesothelioma relative risk (1.01, mean value).

As shown in Table 4, the SHR estimated through competing risks models were lower than the cause‐specific HR, suggesting that the clinical impact of the association between EBRT and mesothelioma was limited due to the high risk of death or incidence of other malignancies. Furthermore, the CIF of mesothelioma after 40 years from the primary diagnosis was very low both among irradiated and nonirradiated subjects (Fig. 2).

**Table 4:** Subhazard ratios of mesothelioma after external beam radiation therapy

	All latency periods				Latency periods between 5 and 10 years^a^				Latency periods of more than 10 years^a^			
	Age‐ and sex‐adjusted estimates		Fully adjusted estimates^b^		Age‐ and sex‐adjusted estimates		Fully adjusted estimates^b^		Age‐ and sex‐adjusted estimates		Fully adjusted estimates^b^	
Site/exposure	SHR	(95% CI)	SHR	(95% CI)	SHR	(95% CI)	SHR	(95% CI)	SHR	(95% CI)	SHR	(95% CI)
Any site												
EBRT												
No	1.00	(Ref.)	1.00	(Ref.)	1.00	(Ref.)	1.00	(Ref.)	1.00	(Ref.)	1.00	(Ref.)
Yes	1.19	(0.94–1.50)	1.25	(0.98–1.59)	1.13	(0.82–1.55)	1.17	(0.85–1.62)	1.36	(0.97–1.93)	1.42	(1.00–2.02)
Pleura												
EBRT												
No	1.00	(Ref.)	1.00	(Ref.)	1.00	(Ref.)	1.00	(Ref.)	1.00	(Ref.)	1.00	(Ref.)
Yes	1.17	(0.91––1.50)	1.24	(0.96–1.61)	1.17	(0.84–1.63)	1.23	(0.87–1.73)	1.26	(0.86–1.84)	1.33	(0.90–1.95)
Pleural irradiation												
None	1.00	(Ref.)	1.00	(Ref.)	1.00	(Ref.)	1.00	(Ref.)	1.00	(Ref.)	1.00	(Ref.)
Scattered	1.26	(0.96–1.65)	1.38	(1.04–1.85)	1.25	(0.88–1.78)	1.36	(0.94–1.96)	1.29	(0.84–1.97)	1.40	(0.90–2.17)
Direct	0.94	(0.61–1.44)	0.95	(0.62–1.47)	0.93	(0.50–1.70)	0.92	(0.50–1.71)	1.18	(0.63–2.22)	1.20	(0.64–2.26)
*P trend*		0.470				0.584		0.519		0.286		0.231
Peritoneum												
EBRT												
No	1.00	(Ref.)	/c	/c	1.00	(Ref.)	/c	/c	1.00	(Ref.)	/c	/c
Yes	1.30	(0.64–2.64)	/c	/c	0.72	(0.22–2.30)	/c	/c	2.13	(0.85–5.36)	/c	/c
Peritoneal irradiation												
None	1.00	(Ref.)	/c	/c	1.00	(Ref.)	/c	/c	1.00	(Ref.)	/c	/c
Scattered	0.55	(0.16–1.88)	/c	/c	/d	/d	/c	/c	/d	/d	/c	/c
Direct	2.15	(0.98–4.75)	/c	/c	1.45	(0.43–4.90)	/c	/c	3.06	(1.12–8.35)	/c	/c
*P trend*		0.171										

95% CI, 95% confidence intervals; EBRT, external beam radiotherapy; Ref., reference category; SHR, subhazard ratio.

a Estimates from regression models including an interaction term between latency and EBRT.

b Estimates adjusted by age, sex, race, year of primary cancer diagnosis, primary cancer surgery, and county's mesothelioma relative risk.

c Analysis not performed due to the limited number of events.

d This category was excluded due to the limited number of cases.

Table S4 presents the estimates for the association between the proxy exposure variable for asbestos and the risk of mesothelioma as a second malignancy. Compared to inhabitants of counties were the RR of primary mesothelioma was below 0.67, patients from high‐risk counties showed a remarkable increase in the incidence of mesothelioma after a primary solid cancer. Indeed, we observed a well‐shaped dose–response, with HR ranging from 2.11 (county's RR of mesothelioma between 0.67 and 0.90) to 5.60 (RR of 1.50 or more).

## Discussion

Our analysis demonstrated that EBRT is a risk factor for mesothelioma. The association was stronger for peritoneal mesothelioma and latency periods longer than 10 years were associated with higher relative risks. We failed to demonstrate a dose–response between radiation dose and pleural mesothelioma risk; the association might be nonlinear and very low doses could still convey an increased risk. A competing risks analysis revealed that the impact of radiation‐induced mesothelioma in our cohort was limited by the high risk of death or other second malignancies. The CIF of mesothelioma after 40 years of observation was extremely low, suggesting that the clinical relevance of radiation‐induced mesothelioma is limited.

Our findings are in line with our previous study that showed an increased risk of mesothelioma in any site (93% of cases were pleural) after EBRT for prostate cancer 6. Although radiation‐induced malignancies are usually expected to occur within the irradiated field (e.g., Baxter et al. 21), even tissues located outside this area are significantly exposed to scattered radiation, as well as to leakage from the radiation source 22, 23, 24. A dose of 1–5 Gy has been estimated for tissues distant 3–10 cm from the irradiated field, while those located more than 10 cm outside the irradiated area can receive doses of 0.1–1 Gy.^14^ These doses are in line with those studied among atomic bomb survivors, when an excess risk of solid cancers was demonstrated for exposure levels as low as 0.1–0.2 Gy 7. In the same population, the risk of solid cancers after radiation exposure showed a linear dose–response only in the range 0–2 Gy, while the function flattened for higher doses 7. Similarly, we did not document a higher risk of pleural mesothelioma for direct exposure compared to scattered irradiation. However, we did observe a considerably increased risk of peritoneal mesothelioma only after direct irradiation, but due to the limited number of cases we cannot rule out the possibility that the large difference in the estimates for scattered and direct radiation was a chance finding. We found that the risk of mesothelioma continues to increase with the latency period, in line with current knowledge on radiation‐induced solid cancers 13.

### Study strength and limitations

To our best knowledge, this is the first study of the dose–response between EBRT and mesothelioma risk in a la large population. Furthermore, we conducted separate analysis for pleural and peritoneal mesothelioma and we studied the role of the latency period. Our study does have limitations. The amount of information on potential confounders was limited and we cannot directly control our estimates for exposure to asbestos – which is always a concern when investigating mesothelioma. Hence, we derived a proxy measure of exposure to asbestos by modeling the RR of primary mesothelioma among males in the county of residence. Despite its obvious limitations, this variable was strongly associated with mesothelioma risk in our population (Table S4). Noteworthy, the estimates of interest (i.e., the HR for EBRT) showed only minor changes after the introduction of the county's RR of mesothelioma in the multivariable models (data not shown). This finding suggests that our estimates were not strongly confounded by asbestos exposure. To further characterize the potential for unmeasured confounding, we conducted a target‐adjustment sensitivity analysis to assess the difference in the prevalence of occupational exposure to asbestos by radiotherapy status necessary to explain the associations observed for latency periods of 10 or more years (Data S1). In the case of pleural mesothelioma, the prevalence of occupational exposure to asbestos should be 55% higher among irradiated subjects compared to nonirradiated (Table S5). Such a large difference seems implausible and, on the balance, we do not believe that unmeasured confounding asbestos exposure can entirely explain our findings. Another limitation of our study is the potential for detection bias, but we observed the higher HR of mesothelioma among irradiated patients after ten or more years from the primary diagnosis. A substantial difference in health monitoring among the studied groups is unlikely to have occurred so far from the primary diagnosis. Confounding by indication (i.e., primary cancer) might affect our estimates. To reduce the potential for this bias, we restricted the analyses to highly informative cancer sites (i.e., those frequently treated with EBRT and with at least one expected case of mesothelioma). Moreover, we explore the presence of within group correlation by fitting shared‐frailty models, but we did not found evidence supporting the assumption of a random intercept. An obvious limitation of our study is the wrong assignment of EBRT exposure in register data; however, this kind of nondifferential misclassification is likely to bias the estimates of interest toward the null hypothesis. Finally, we could not investigate extremely long latency periods due to the limited time period covered by the SEER registries: our cohort included only eleven secondary mesothelioma cases occurred more than 20 years after the primary diagnosis. Hence, our study provides information only for relatively short (<20 years) latency periods. As asbestos‐related mesothelioma is usually diagnosed decades after the first exposure, 1 we cannot rule out the hypothesis that the risk of radiation‐induced mesothelioma might continue to increase for several years after the exposure to EBRT.

## Conclusions

Our study shows that exposure to ionizing radiation – in particular to EBRT ‐ might be a determinant of mesothelioma. Longer latency periods are associated with higher relative risks, whereas the dose–response relationship seems be nonlinear. Despite the etiological association, the clinical impact of secondary mesothelioma after EBRT for a primary solid cancer is limited.

## Conflict of interest

The authors declare that they have no conflict of interest.

## Supporting information


**Figure S1.** Standardized incidence ratios (SIR) of mesothelioma among males aged between 20 and 84 years old.

**Figure S2.** Relative risk (RR) of mesothelioma among men estimated with Besag‐York‐Mollier models. SEER 13 registries (excluding Alaska natives register), 1992–2012.

**Table S1.** Expected cases of mesothelioma by external beam radiotherapy status among cancer 968,070 patients (see Fig. 1).

**Table S2.** Characteristics of the study population by primary cancer site.

**Table S3.** Cause‐specific hazard ratios of peritoneal mesothelioma (all latency periods)

**Table S4.** Risk of mesothelioma (any site) as a second malignancy by RR of primary mesothelioma among males in the county of residence. Cause‐specific hazard ratios from Cox proportional hazards regression models.

**Table S5.** Target‐adjustment sensitivity analysis. Estimated prevalence of occupational exposure to asbestos necessary to explain the observed association between mesothelioma and external beam radiotherapy.

**Data S1.** Radiation‐induced mesothelioma among long‐term solid cancer survivors: a longitudinal analysis of SEER database.
