# Supplementary figures and images for: Radiation‐induced mesothelioma among long‐term solid cancer survivors: a longitudinal analysis of SEER database
Source: Cancer Med. 2016 Feb 10;5(5):950–9. doi: 10.1002/cam4.656 (PMC4864824; doi:10.1002/cam4.656)

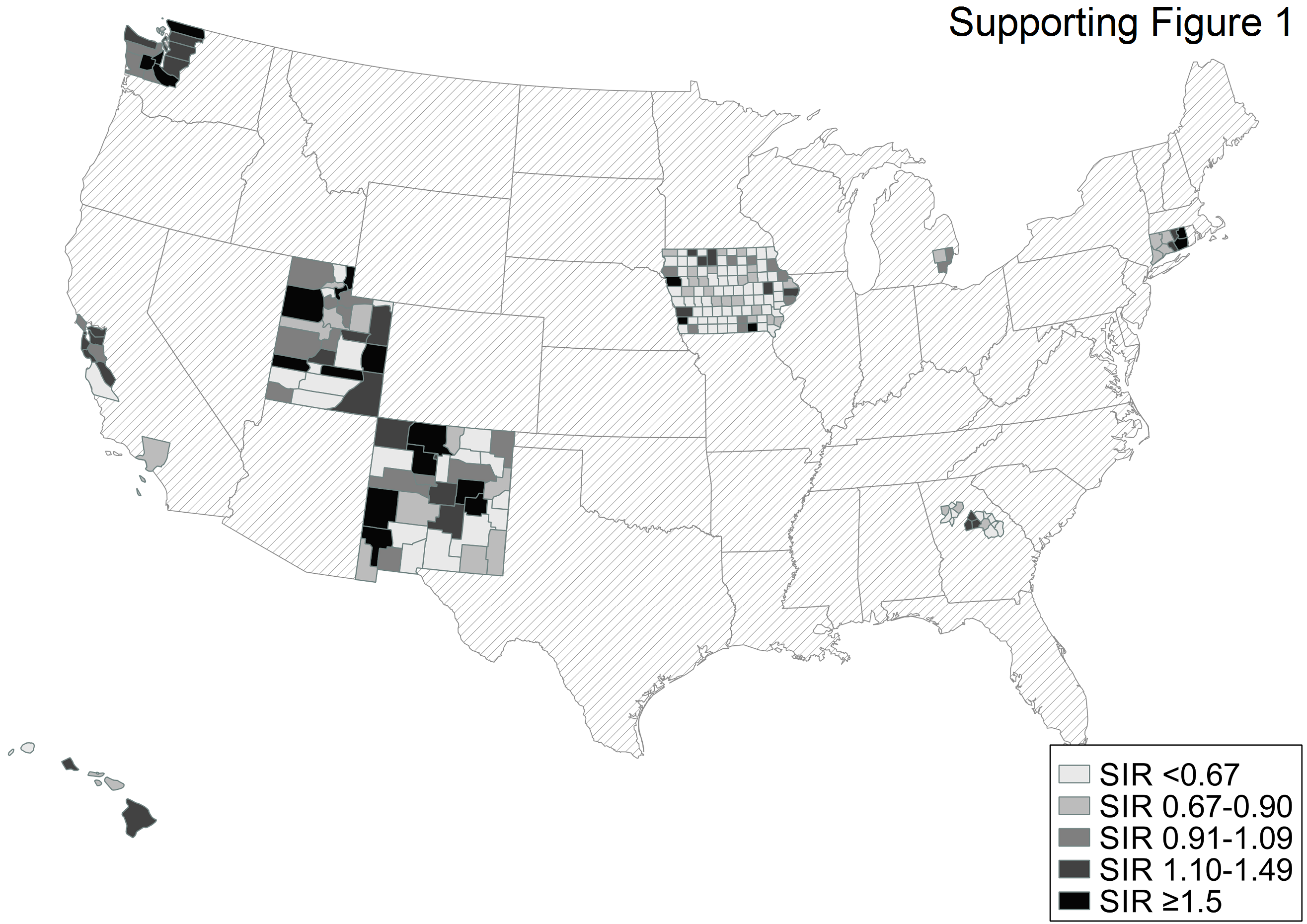

Supplement: Supplementary file 1 — Figure S1. Standardized incidence ratios (SIR) of mesothelioma among males aged between 20 and 84 years old. [file CAM4-5-950-s001.tif]

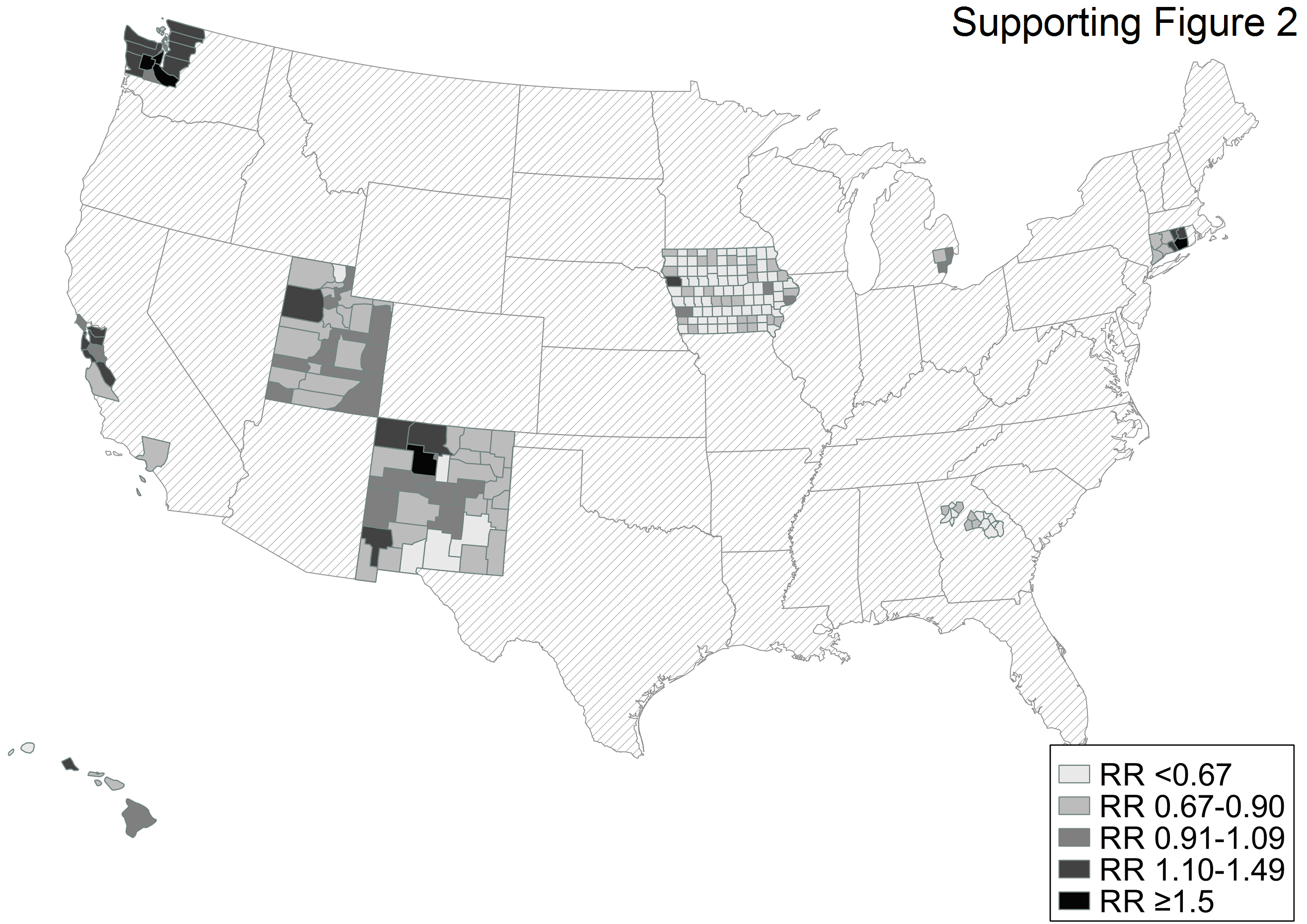

Supplement: Supplementary file 2 — Figure S2. Relative risk (RR) of mesothelioma among men estimated with Besag‐York‐Mollier models. SEER 13 registries (excluding Alaska natives register), 1992–2012. [file CAM4-5-950-s002.tif]
