# Supplementary material for: Radiation‐induced mesothelioma among long‐term solid cancer survivors: a longitudinal analysis of SEER database
Source: Cancer Med. 2016 Feb 10;5(5):950–9. doi: 10.1002/cam4.656 (PMC4864824; doi:10.1002/cam4.656)
Supplement: Supplementary file 3 — Table S1. Expected cases of mesothelioma by external beam radiotherapy status among cancer 968,070 patients (see Fig. 1). [file CAM4-5-950-s003.docx]

**Supporting Table 1.** Expected cases of mesothelioma by external beam radiotherapy status among cancer 968,070 patients (see Figure 1). Expected numbers were computed based on the gender-, age- (5-years classes), race- (white/black/other), and calendar year- (5-years periods) specific rates of mesothelioma in the SEER registries (1973/2009).

|  |  | **External beam radiotherapy** | | |  |
| --- | --- | --- | --- | --- | --- |
| **Primary cancer site** | **Patients**  **(N=968,070)** | **All** | **Yes** | **No** | **Site included^a^** |
| Prostate | 311,548 | 198.33 | 136.19 | 62.14 | Yes |
| Breast | 343,068 | 40.29 | 25.82 | 14.48 | Yes |
| Rectum and rectosigmoid junction | 50,061 | 19.79 | 14.68 | 5.11 | Yes |
| Lung and bronchus | 46,382 | 13.51 | 11.16 | 2.35 | Yes |
| Oral cavity and pharynx | 36,964 | 12.99 | 8.50 | 4.49 | Yes |
| Corpus and uteros NOS | 69,717 | 11.14 | 8.22 | 2.92 | Yes |
| Larynx | 15,426 | 8.26 | 2.43 | 5.83 | Yes |
| Stomach | 10,385 | 3.21 | 2.86 | 0.35 | Yes |
| Testis | 20,659 | 2.96 | 1.19 | 1.77 | Yes |
| Cervix uteri | 26,800 | 2.32 | 1.41 | 0.91 | Yes |
| Penis and other male genital organs | 1,824 | 1.09 | 1.01 | 0.08 | Yes |
| Eye and orbit | 2,803 | 1.02 | 0.89 | 0.13 | Yes |
| Anus, anal canal and anorectum | 4,416 | 0.99 | 0.38 | 0.61 | No |
| Esophagus | 2,873 | 0.88 | 0.51 | 0.38 | No |
| Other respiratory organs | 2,600 | 0.75 | 0.40 | 0.35 | No |
| Ureter and other urinary organs | 1,563 | 0.73 | 0.67 | 0.06 | No |
| Gallbladder and other biliary | 2,243 | 0.60 | 0.52 | 0.09 | No |
| Brain | 8,083 | 0.60 | 0.27 | 0.33 | No |
| Vulva | 4,399 | 0.49 | 0.46 | 0.03 | No |
| Pancreas | 2,552 | 0.49 | 0.38 | 0.11 | No |
| Other female genital organs | 2,268 | 0.21 | 0.15 | 0.06 | No |
| Thymus | 1,015 | 0.16 | 0.07 | 0.09 | No |
| Adrenal gland | 421 | 0.08 | 0.07 | 0.00 | No |

Abbreviations: NOS, not otherwise specified

^a^Site with less than one expected case were excluded from the final study population
