# Supplementary material for: Radiation‐induced mesothelioma among long‐term solid cancer survivors: a longitudinal analysis of SEER database
Source: Cancer Med. 2016 Feb 10;5(5):950–9. doi: 10.1002/cam4.656 (PMC4864824; doi:10.1002/cam4.656)
Supplement: Supplementary file 4 — Table S2. Characteristics of the study population by primary cancer site. [file CAM4-5-950-s004.docx]

**Supporting Table 2.** Characteristics of the study population by primary cancer site.

| **Characteristic** | **Eye and orbit**  (n=2,803) | **Oral cavity and pharynx**  (n=36,964) | **Larynx**  (n=15,426) | **Lung and bronchus**  (n=46,382) | **Stomach**  (n=10,385) | **Rectum and**  **rectosigmoid junction**  (n=50,061) | **Breast**  (n=343,068) | **Cervix uteri**  (n=26,800) | **Corpus and uterus NOS**  (n=69,717) | **Prostate**  (n=311,548) | **Testis**  (n=20,659) | **Penis and other**  **male genital organs**  (n=1,824) |
| --- | --- | --- | --- | --- | --- | --- | --- | --- | --- | --- | --- | --- |
| Age (years), mean | 55.5 | 56.4 | 60.1 | 61.9 | 61.7 | 61.2 | 57.3 | 45.0 | 59.4 | 66.0 | 34.6 | 59.8 |
| Gender |  |  |  |  |  |  |  |  |  |  |  |  |
| -female, % | 44.8 | 33.3 | 18.5 | 48.8 | 42.2 | 44.2 | 99.4 | 100.0 | 100.0 | 0.0 | 0.0 | 0.0 |
| -male, % | 55.2 | 66.7 | 81.5 | 51.2 | 57.8 | 55.8 | 0.6 | 0.0 | 0.0 | 100.0 | 100.0 | 100.0 |
| Race |  |  |  |  |  |  |  |  |  |  |  |  |
| -white, % | 94.6 | 83.4 | 84.4 | 83.7 | 64.3 | 82.0 | 84.1 | 76.4 | 87.6 | 80.8 | 92.8 | 82.1 |
| -black, % | 1.5 | 6.6 | 11.2 | 9.2 | 11.9 | 6.8 | 7.4 | 11.8 | 4.4 | 12.1 | 2.1 | 8.5 |
| -other^a^, % | 3.9 | 10.0 | 4.4 | 7.1 | 23.8 | 11.2 | 8.4 | 11.8 | 7.9 | 7.1 | 5.2 | 9.4 |
| Year of diagnosis |  |  |  |  |  |  |  |  |  |  |  |  |
| -1973-1977, % | 13.6 | 9.7 | 11.8 | 7.6 | 8.1 | 8.6 | 6.9 | 11.6 | 12.1 | 2.9 | 5.5 | 11.3 |
| -1978-1982, % | 13.7 | 11.2 | 13.7 | 10.2 | 9.5 | 10.0 | 7.7 | 10.6 | 10.6 | 4.0 | 8.3 | 11.1 |
| -1983-1987, % | 10.7 | 11.5 | 13.7 | 11.6 | 9.8 | 11.6 | 10.1 | 10.8 | 10.9 | 5.6 | 10.8 | 12.3 |
| -1988-1992, % | 13.1 | 12.4 | 15.3 | 13.6 | 12.4 | 12.8 | 13.4 | 14.6 | 12.5 | 14.3 | 13.9 | 13.4 |
| -1993-1997, % | 17.4 | 17.1 | 16.7 | 18.2 | 17.4 | 16.5 | 19.1 | 19.5 | 17.1 | 22.7 | 19.2 | 18.1 |
| -1998-2002, % | 15.4 | 18.1 | 15.3 | 18.8 | 20.1 | 20.2 | 22.0 | 18.1 | 18.2 | 24.9 | 21.0 | 17.4 |
| -2003-2007, % | 16.1 | 20.1 | 13.6 | 20.0 | 22.7 | 20.4 | 20.9 | 14.9 | 18.7 | 25.6 | 21.3 | 16.3 |
| Cancer-directed surgery |  |  |  |  |  |  |  |  |  |  |  |  |
| -no, % | 21.2 | 22.8 | 41.3 | 19.4 | 8.1 | 3.9 | 1.8 | 22.4 | 3.5 | 42.9 | 1.7 | 4.4 |
| -yes, % | 78.8 | 77.2 | 58.7 | 80.6 | 91.9 | 96.1 | 98.2 | 77.6 | 96.5 | 57.1 | 98.3 | 95.6 |
| EBRT |  |  |  |  |  |  |  |  |  |  |  |  |
| -no, % | 81.9 | 52.1 | 26.2 | 76.2 | 84.9 | 69.5 | 56.4 | 64.1 | 75.4 | 68.3 | 55.6 | 92.3 |
| -yes, % | 18.1 | 47.9 | 73.8 | 23.8 | 15.1 | 30.5 | 43.6 | 35.9 | 24.6 | 31.7 | 44.4 | 7.7 |

Abbreviation: EBRT, external beam radiotherapy

^a^Other includes American Indian/AK Native; Asian/Pacific Islander; other unspecified; unknown.
