# Supplementary material for: Radiation‐induced mesothelioma among long‐term solid cancer survivors: a longitudinal analysis of SEER database
Source: Cancer Med. 2016 Feb 10;5(5):950–9. doi: 10.1002/cam4.656 (PMC4864824; doi:10.1002/cam4.656)
Supplement: Supplementary file 5 — Table S3. Cause‐specific hazard ratios of peritoneal mesothelioma (all latency periods) [file CAM4-5-950-s005.docx]

**SupportingTable 3.** Cause-specific hazard ratios of peritoneal mesothelioma (all latency periods)

|  | **Model** | | | | | | | | | | | | | | | |
| --- | --- | --- | --- | --- | --- | --- | --- | --- | --- | --- | --- | --- | --- | --- | --- | --- |
|  | **Univariate** | | **Adjusted by age** | | **Adjusted by sex** | | **Adjusted by race** | | **Adjusted by year of primary cancer** | | **Adjusted by primary cancer surgery** | | **Adjusted by county’s mesothelioma relative risk.** | | **Adjusted by age- and sex** | |
| **Peritoneal irradiation** | *HR* | *(95%CI)* | *HR* | *(95%CI)* | *HR* | *(95%CI)* | *HR* | *(95%CI)* | *HR* | *(95%CI)* | *HR* | *(95%CI)* | *HR* | *(95%CI)* | *HR* | *(95%CI)* |
| -none | 1.00 | (Ref.) | 1.00 | (Ref.) | 1.00 | (Ref.) | 1.00 | (Ref.) | 1.00 | (Ref.) | 1.00 | (Ref.) | 1.00 | (Ref.) | 1.00 | (Ref.) |
| -scattered | 0.56 | (0.16-1.88) | 0.61 | (0.18-2.08) | 0.61 | (0.18-2.09) | 0.56 | (0.16-1.88) | 0.54 | (0.16-1.85) | 0.56 | (0.16-1.88) | 0.55 | (0.16-5.28) | 0.64 | (0.18-2.21) |
| -direct | 2.46 | (1.14-5.30) | 2.28 | (1.05-4.94) | 2.28 | (1.03-5.04) | 2.49 | (1.15-5.37) | 2.44 | (1.13-5.26) | 2.47 | (1.07-5.72) | 2.45 | (1.14-5.28) | 2.20 | (0.99-4.88) |
| *P trend* |  | 0.064 |  | 0.086 |  | 0.101 |  | 0.061 |  | 0.067 |  | 0.109 |  | 0.066 |  | 0.114 |

Abbreviations: 95%CI, 95% confidence intervals; EBRT, external beam radiotherapy; HR, hazard ratio; Ref., reference category.
