# Supplementary material for: Radiation‐induced mesothelioma among long‐term solid cancer survivors: a longitudinal analysis of SEER database
Source: Cancer Med. 2016 Feb 10;5(5):950–9. doi: 10.1002/cam4.656 (PMC4864824; doi:10.1002/cam4.656)
Supplement: Supplementary file 6 — Table S4. Risk of mesothelioma (any site) as a second malignancy by RR of primary mesothelioma among males in the county of residence. Cause‐specific hazard ratios from Cox proportional hazards regression models. [file CAM4-5-950-s006.docx]

**Supporting Table 4.** Risk of mesothelioma (any site) as a second malignancy by RR of primary mesothelioma among males in the county of residence. Cause-specific hazard ratios from Cox proportional hazards regression models.

|  | **Case** | | **Age- and sex-adjusted**  **estimates** | | **Fully adjusted**  **Estimates^a^** | |
| --- | --- | --- | --- | --- | --- | --- |
| **County’s RR of mesothelioma** | *Yes* | *No* | *HR* | *(95%CI)* | *HR* | *(95%CI)* |
| - <0.67 | 12 | 96,852 | 1.00 | (Ref.) | 1.00 | (Ref.) |
| - 0.67-0.90 | 89 | 356,779 | 2.18 | (1.19-3.99) | 2.11 | (1.15-3.86) |
| - 0.91-1.09 | 61 | 198,412 | 2.52 | (1.36-4.68) | 2.47 | (1.33-4.59) |
| - 1.10-1.49 | 113 | 245,962 | 3.59 | (1.98-6.51) | 4.01 | (2.21-7.29) |
| - ≥1.50 | 26 | 37,331 | 5.72 | (2.88-11.3) | 5.60 | (2.82-11.1) |
| *P trend* |  |  |  | <0.001 |  | <0.001 |

Abbreviations: 95%CI, 95% confidence intervals; HR, hazard ratio; Ref., reference category.

^a^Estimates adjusted by age, sex, race, year of primary cancer diagnosis, primary cancer surgery, and external beam radiotherapy.
