# Supplementary material for: Radiation‐induced mesothelioma among long‐term solid cancer survivors: a longitudinal analysis of SEER database
Source: Cancer Med. 2016 Feb 10;5(5):950–9. doi: 10.1002/cam4.656 (PMC4864824; doi:10.1002/cam4.656)
Supplement: Supplementary file 7 — Table S5. Target‐adjustment sensitivity analysis. Estimated prevalence of occupational exposure to asbestos necessary to explain the observed association between mesothelioma and external beam radiotherapy. [file CAM4-5-950-s007.docx]

**Suppporting 5.** Target-adjustment sensitivity analysis. Estimated prevalence of occupational exposure to asbestos necessary to explain the observed association between mesothelioma and external beam radiotherapy.

| **Scenario A: overall prevalence of exposure to asbestos equal to 20%** | | | | | | | |
| --- | --- | --- | --- | --- | --- | --- | --- |
| **Outcome** | **Estimate**  **(see Table 1)** | **HR** | **RR associated with asbestos** | **Prevalence of exposure to asbestos by EBRT status** | | | |
|  |  |  |  | **Exposed** | **Unexposed** | **Difference** | **Ratio** |
| Mesothelioma in any site | EBRT vs no EBRT  (latency >10 years) | 1.58 | 53.4 | 27.0% | 16.4% | 10.6% | 1.65 |
| Plerual mesothelioma | EBRT vs no EBRT  (latency >10 years) | 1.49 | 53.4 | 26.0% | 16.8% | 9.2% | 1.55 |
| Peritoneal mesothelioma | EBRT vs no EBRT  (latency >10 years) | 2.31 | 53.4 | 33.0% | 13.2% | 19.8% | 2.50 |
| **Scenario B: overall prevalence of exposure to asbestos equal to 40%** | | | | | | | |
| **Outcome** | **Estimate**  **(see Table 1)** | **HR** | **RR associated with asbestos** | **Prevalence of exposure to asbestos by EBRT status** | | | |
|  |  |  |  | **Exposed** | **Unexposed** | **Difference** | **Ratio** |
| Mesothelioma in any site | EBRT vs no EBRT  (latency >10 years) | 1.58 | 27.2 | 53.9% | 32.7% | 21.2% | 1.65 |
| Plerual mesothelioma | EBRT vs no EBRT  (latency >10 years) | 1.49 | 27.2 | 52.1% | 33.7% | 18.4% | 1.55 |
| Peritoneal mesothelioma | EBRT vs no EBRT  (latency >10 years) | 2.31 | 27.2 | 47.7% | 26.4% | 39.6% | 2.50 |

Abbreviations: EBRT, external beam radiotherapy; HR, hazard ratio.
