# Supplementary material for: Radiation‐induced mesothelioma among long‐term solid cancer survivors: a longitudinal analysis of SEER database
Source: Cancer Med. 2016 Feb 10;5(5):950–9. doi: 10.1002/cam4.656 (PMC4864824; doi:10.1002/cam4.656)
Supplement: Supplementary file 8 — Data S1. Radiation‐induced mesothelioma among long‐term solid cancer survivors: a longitudinal analysis of SEER database. [file CAM4-5-950-s008.docx]

**Radiation-induced mesothelioma among long-term solid cancer survivors: a longitudinal analysis of SEER database**

**Supporting Resource 1.**

**Relative risk of mesothelioma by county**

Relative risk (RR) of primary mesothelioma by county was estimated as a proxy of individual exposure to asbestos. This analysis was based on primary mesothelioma cases among males aged between 20 and 84 years old reported to the SEER 13 Registries between 1992 and 2012. At first, we used the entire population covered by the SEER 13 registers to estimate the mesothelioma rates specific by sex, age (5-years classes), race (black, white, other), and calendar period (1992-1994, 1995-1999, 2000-2004, 2005-2009, 2010-2012). Second, we calculated the expected number of cases by county by applying the rates to the population numbers provided in SEER*Stat 8.2.1. The standardized incidence ratios (SIR) of mesothelioma where then calculated as the ratio between the observed and the expected cases. Web Figure 1 presents the SIR for the 214 studied counties. On average, Seattle-Puget Sound was the registry with the highest SIR, while the registries for Hawaii, Atlanta and Rural Georgia had the lowest standardized incidence. The Iowa registry includes 99 counties, 89 of which had less than 10 expected cases of mesothelioma; the high degree of fragmentation of the Iowa registry resulted in substantial heterogeneity, which is reflected in the SIR data.

*The Besag-York-Molliè model*

Disease mapping based on SIRs presents two important issues:

- artifacts (e.g. SIR equal to zero or assuming extreme values) may occur in the presence of small expected numbers;
- the spatial structure of the data is ignored.

To solve these problems, the observed and the expected counts can be fitted in a conditional autoregressive Bayesian model that accounts for heterogeneity and clustering. For this purposes, the most widely used is the Besag-York-Mollè (BYM) model [1a]. Based on the assumption that neighboring areas share similar characteristics, this model includes a random effect term for spatial adjacency; thus, it forces the area-specific estimates toward a local mean. The BYM model also includes a random effect term to account for unstructured sources of error (heterogeneity). The Bayesian framework in which the BYM model is implemented implies the specification of hyper-prior distributions for the parameters of the two random effects.

*Parameter estimation*

The BYM model for the RR of mesothelioma was fitted using WinBugs 1.4.3 that implements the Markov Chain Monte Carlo method. We assumed a “vague” hyper-prior for the random effect terms and the adjacency matrix of the counties was based on contiguity. We joined two counties (namely Valencia County and Cibola County, NM), as they were prior to 1981. After a burn-in of 90,000 iterations (i.e. 90,000 iterations were discarded to ensure convergence), the posterior distribution of the RRs was derived from 10,000 iterations. The estimated RRs are presented in Supporting Figure 2. The results of the BYM model differ from the simple SIR analysis (Supporting Figure 1) in that a lower degree of heterogeneity was appreciable in Iowa and New Mexico. Also, RRs estimated for counties of New Mexico apparently presented a gradient increasing from the south-eastern corner to the north-western corner.

To evaluate if the county’s RR of primary mesothelioma was able to predict the risk of secondary mesothelioma in our cohort, we estimated the cause-specific hazard ratios of mesothelioma (Supporting Table 2). The variable that we constructed as a proxy of asbestos exposure showed a strong association with mesothelioma, also demonstrating a well-shaped exposure-response relationship.

**Target-adjustment sensitivity analysis**

Target-adjustment sensitivity analysis is used to calculate the magnitude of a single bias necessary for the adjusted estimate to be a certain value (typically the null) [2a]. We applied this method to simulate the difference in prevalence of occupational exposure to asbestos necessary to change to 1.00 the incidence rate ratio (IRR) of mesothelioma observed for subjects exposed to external beam radiotherapy (EBRT) compared to subjects who did not receive radiotherapy.

*Methods*

Formulas

Assuming the absence of interactions, the following formula can be used (derived from Lyn and colleagues [3a]):

$\frac{\text{RR}_{\text{asbestos}}\times\Pr(Z^{+}|E^{+})+(1-\Pr(Z^{+}|E^{+}))}{\text{RR}_{\text{asbestos}}\times\Pr(Z^{+}|E^{-})+(1-\Pr(Z^{+}|E^{-}))}\times RR_{t\arg et}=RR_{observed}$ (eq. 1)

where E is the exposure of interest (i.e. EBRT), Z is an unmeasured confounder (i.e. asbestos), RR_asbestos_ is the RR of diseases associated with the confounder, RR_target_ is target of the analysis (i.e. RR = 1), and RR_observed_ is the observed RR of disease associated with the exposure. Establishing an RR_target_ equal to 1, it is possible to solve (eq. 1) for Pr(Z^+^|E^+^) as:

$\Pr(Z^{+}|E^{+})=\frac{RR_{observed}(\Pr(Z^{+}|E^{-})\times(RR_{asbestos}-1)+1)-1}{RR_{asbestos}-1}$ (eq. 2)

The overall prevalence of the exposure to the confounder Z can be written as a function of the prevalence in each stratum of the exposure E:

$\Pr(Z^{+})=\Pr(Z^{+}|E^{+})\times\Pr\left(E^{+}\right)+\Pr(Z^{+}|E^{-})\times\Pr\left(E^{-}\right)$ (eq. 3)

thus

$\Pr(Z^{+}|E^{-})=\frac{\Pr(Z^{+})-\Pr(Z^{+}|E^{+})\times\Pr(E^{+})}{\Pr(E^{-})}$ (eq. 4)

or

$\Pr(Z^{+}|E^{+})=\frac{\Pr(Z^{+})-\Pr(Z^{+}|E^{-})\times\Pr(E^{-})}{\Pr(E^{+})}$ (eq. 5)

Substituting (eq. 4) in (eq. 2) we obtain:

$\Pr(Z^{+}|E^{-})=\frac{\frac{\Pr(Z^{+})\times(RR_{asbestos}-1)}{\Pr(E^{+})}+1-RR_{observed}}{\frac{\Pr(E^{-})\times(RR_{asbestos}-1)}{\Pr(E^{+})}+RR_{observed}(RR_{asbestos}-1)}$ (eq. 6)

After calculating Pr(Z^+^|E^-^) we can obtain Pr(Z^+^|E^+^) from (eq. 5).

Assumptions

Reports on the prevalence of occupational exposure to asbestos in the general population were highly variable, ranging from about 15% to more than 40% [4-8]. In the absence of data directly applicable to the SEER registries, we performed our calculation using two different lifelong probabilities of occupational exposure to asbestos: 20% and 40%. We estimated the risk of mesothelioma for subjects exposed to asbestos based on data from the SEER 9 registries database and the SEER 13 registries database. We only used data for subjects aged above twenty years, to increase the comparability with our study population. During the study period (1973-2012), 8,140 cases of mesothelioma were observed in 679,783,279 person-years. Applying a background rate of mesothelioma (i.e. mesotheliomas not associated with asbestos exposure) of 1 per 1.000.000 person-years [9], 857 cases would have been expected. Hence, we estimated that 8,984 cases could be attributed to asbestos exposures, corresponding to a population attributable fraction (PAF) of 91.3% (in line with current knowledge [10]).

Assuming the absence of confounding, the PAF can be calculated as follows [11]:

$PAF=\frac{\Pr(E^{+})(RR-1)}{1+\Pr(E^{+})(RR-1)}$ (eq. 7)

Solving by RR we obtain:

$RR=\frac{PAF\times\Pr(E^{+})-PAF-\Pr(E^{+})}{PAF\times\Pr(E^{+})-\Pr(E^{+})}$ (eq. 8)

Using (eq. 8) we can calculate a RR of mesothelioma associated with asbestos of 53.4 for a prevalence of exposure of 20%, and a RR of 27.2 for a prevalence of 40%.

*Results*

Results from the target-adjustment sensitivity analysis are presented in Supporting Table 4. We calculated the ratio between the proportion of subjects exposed to asbestos among EBRT patients and the proportion of exposed to asbestos in the reference group. An exposure ratio of 1.67 would be necessary to explain completely the IRR of mesothelioma observed for EBRT when studying latency periods of 10 years or more. Furthermore, the ratio should be as high as 2.50 to explain the point estimate observed for peritoneal mesothelioma after 10 years from the primary diagnosis.

**References**

1. Besag J, York J, Mollie A. Bayesian image restoration, with two applications in spatial statistics (with discussion). Ann Inst Stat Math. 1991;43:1-59.
2. Philips CV. Quantifying and reporting uncertainty from systematic errors. Epidemiology. 2006;14:459-66.
3. Lin DY, Psaty BM, Kronmal RA. Assessing the sensitivity of regression results to unmeasured confounders in observational studies. Biometrics. 1998;54:948-63.
4. De Matteis S, Consonni D, Lubin JH, et al. Impact of occupational carcinogens on lung cancer risk in a general population. Int J Epidemiol. 2012;41:711-21.
5. Garabrant DH, Peters RK, Homa DM. Asbestos and colon cancer: lack of association in a large case-control study. Am J Epidemiol. 1992;135:843-53.
6. Spirtas R, Heineman EF, Bernstein L, et al. Malignant mesothelioma: attributable risk of asbestos exposure. Occup Environ Med. 1994;51:804-11.
7. Berrino F, Richiardi L, Boffetta P, et al. Occupation and larynx and hypopharynx cancer: a job-exposure matrix approach in an international case-control study in France, Italy, Spain and Switzerland. Cancer Causes Control. 2003;14:213-23.
8. Pohlabeln H, Wild P, Schill W, et al. Asbestos fibreyears and lung cancer: a two phase case-control study with expert exposure assessment. Occup Environ Med. 2002;59:410-4.
9. Teta MJ, Mink PJ, Lau E, Sceurman BK, Foster ED. US mesothelioma patterns 1973-2002: indicators of change and insights into background rates. Eur J Cancer Prev. 2008;17:525-34.
10. Rushton L, Bagga S, Bevan R, et al. Occupation and cancer in Britain. Br J Cancer 2010;102:1428-37.
11. Porta MS, Greenland S, Hernán M, dos Santos Silva I, Last JM. A dictionary of epidemiology. New York: Oxford University Press, 2014:13
